# Treatment of post-intubation laryngeal granulomas: systematic review and proportional meta-analysis^☆^

**DOI:** 10.1016/j.bjorl.2018.03.003

**Published:** 2018-04-14

**Authors:** Caroline Fernandes Rimoli, Regina Helena Garcia Martins, Daniele Cristina Catâneo, Rui Imamura, Antonio José Maria Catâneo

**Affiliations:** a Universidade Estadual Paulista (Unesp), Faculdade de Medicina de Botucatu, Departamento de Oftalmologia, Otorrinolaringologia e Cirurgia de Cabeça e Pescoço, Botucatu, SP, Brazil; b Universidade Estadual Paulista (Unesp), Faculdade de Medicina de Botucatu, Departamento de Cirurgia, Botucatu, SP, Brazil; c Universidade de São Paulo (USP), Departamento de Otorrinolaringologia, São Paulo, SP, Brazil

**Keywords:** Granulomas, Larynx, Treatment, Systematic review, Granulomas, Laringe, Tratamento, Revisão sistemática

## Abstract

**Introduction:**

Laryngeal granulomas post intubation are benign but recurrent lesions. There is no consensus for its treatment.

**Objective:**

To describe the effectiveness of different treatment modalities for primary or recurrent laryngeal granulomas resulting from endotracheal intubation.

**Methods:**

Systematic review and proportional meta-analysis. Eligibility criteria – experimental or observational studies with at least five subjects. Outcomes studied – granuloma resolution, recurrence, and time for resolution. Databases used – Pubmed, Embase, Lilacs, and Cochrane. The Stats Direct 3.0.121 program was used.

**Results:**

Six studies were selected, with 85 patients. The treatments registered were: antireflux therapy, speech therapy, anti-inflammatory drugs, steroids, antibiotics, zinc sulfate and surgery. 85 patients from six studies had primary treatment: surgery ± associations (41 patients), resolution chance 75% (95% CI: 0.3–100%, *I*^2^ = 90%), absolute relapse risk 25% (95% CI: 0.2–71%); medical treatment (44 patients), resolution chance 86% (95% CI: 67–97%); and absolute relapse risk 14% (95% CI: 3–33%). There was no significant difference between groups. Three studies, encompassing 19 patients, analyzed secondary treatment (failure or recurrence after primary treatment); three subjects presented new recurrence. The time needed to resolve the lesions varied from immediate, after surgery, to 23 months, for inhaled steroid.

**Conclusion:**

There is no evidence of high quality that proves the efficacy of any treatment for laryngeal granulomas resulting from endotracheal intubation.

## Resumo

**Introdução:**

Granulomas laríngeos pós-intubação são lesões laríngeas benignas, porém recorrentes. Não há um consenso na literatura em relação ao seu tratamento.

**Objetivo:**

Descrever a eficácia de diferentes modalidades de tratamento para granulomas laríngeos primários ou recorrentes resultantes da intubação endotraqueal.

**Método:**

Estudo-revisão sistemática e metanálise proporcional. Critérios de elegibilidade: estudos experimentais ou observacionais com pelo menos cinco indivíduos. Desfechos estudados: resolução do granuloma, recorrência e tempo de resolução. Bases de dados usadas: Pubmed, Embase, Lilacs e Cochrane. Foi usado o *software* Stats Direct 3.0.121.

**Resultados:**

Foram selecionados seis estudos, com 85 pacientes. Os tratamentos registrados foram: terapia antirrefluxo, terapia da voz, medicamentos anti-inflamatórios, esteroides, antibióticos, sulfato de zinco e cirurgia. Receberam tratamento primário 85 pacientes de seis estudos: cirurgia ± associações (41 pacientes), chance de resolução de 75% (IC 95% 0,3% a 100%, I^2^ = 90%) e risco absoluto de recorrência de 25% (IC 95%: 0,2% a 71%); tratamento clínico (44 pacientes), chance de resolução de 86% (IC 95%: 67% a 97%) e risco absoluto de recorrência de 14% (IC 95%: 3% a 33%). Não houve diferença significante entre os grupos. Três estudos, que abrangeram 19 pacientes, analisaram o tratamento secundário (falha ou recorrência após o tratamento primário); três indivíduos apresentaram nova recorrência. O tempo necessário para resolver as lesões variou de imediato, logo após a cirurgia, até 23 meses, com tratamento com esteroides inalados.

**Conclusão:**

Não há evidências de alta qualidade que provem a eficácia de qualquer tratamento para granulomas laríngeos resultantes da intubação endotraqueal.

## Introduction

**Figure 1:**
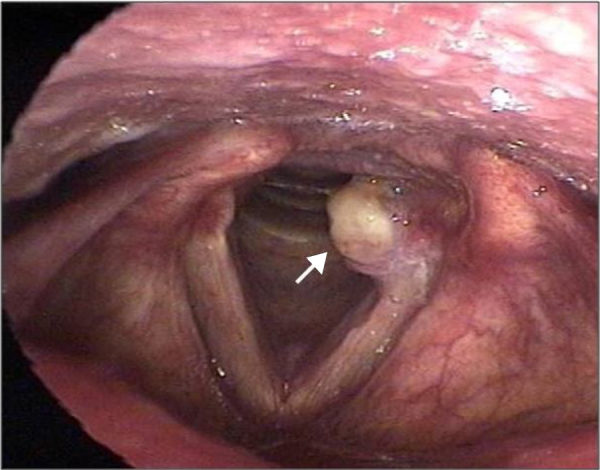
Vocal fold granuloma (arrow).

Laryngeal granulomas are benign, non-neoplastic, uni- or bilateral lesions that develop in the posterior third of the vocal folds, more specifically in the vocal process or in the arytenoid region (Fig. 1). They represent a repairing, reactive inflammatory process of the local mucosa, secondary to local injury, ulceration, and exposure of the perichondrium of the arytenoid cartilage. Histology shows an infiltrate of polymorphonuclear inflammatory cells and lymphocytes, permeated by blood lakes. They may be sessile or pedunculated, with predominance between the fourth and fifth decades of life.^1^, ^2^, ^3^, ^4^

The etiology of laryngeal granulomas is multifactorial: vocal abuse (33%),^1^, ^5^ traumatic and prolonged endotracheal intubation (23%),^6^, ^7^, ^8^, ^9^ and gastroesophageal reflux (30%)^10^, ^11^, ^12^ are most commonly involved. Chronic cough is another possible cause of laryngeal granulomas. When the etiology cannot be determined, the granulomas are considered of idiopathic origin. Among all causes, endotracheal intubation is highly causal due to the high recurrence rate of such lesions.^12^

The treatment of granulomas depends directly on the etiology. In granulomas secondary to gastroesophageal reflux, the mucosa of the pharynx and larynx are exposed to acid content and pepsin from the stomach, as well as bile and pancreatic enzymes, maintaining a constant inflammatory process, evolving to edema, inflammation, ulceration and granuloma. In these granulomas, there is evident improvement of the symptoms and regression of the lesions with dietary treatment associated with proton pump inhibitors.^12^, ^13^, ^14^ In granulomas due to vocal abuse, speech-language therapy with voice re-education is advantageous.^5^ However, a favorable progression of these treatments is not the usual course of post-intubation granulomas. In intubated patients, the cannula is positioned in the posterior region of the glottis, between the vocal processes of the arytenoid cartilages, what can cause local scarification and damage to the cartilages, resulting in granuloma formation. The situation is further aggravated by the use of larger diameter tubes, superficial sedation, and traumatic and prolonged intubation, longer than 10–12 days.^4^, ^6^, ^7^, ^8^, ^9^

The recommended treatments for post intubation granulomas include voice therapy,^5^ antireflux drugs,^12^, ^13^, ^14^, ^15^, ^16^ surgery,^17^, ^18^ botulin toxin,^19^, ^20^, ^21^ zinc sulphate,^22^ mitomycin C,^23^, ^24^ and steroids.^25^, ^26^

Zhang et al.^18^ evaluated the efficacy of surgery as compared to conservative treatment of laryngeal granulomas in 13 patients, six of whom underwent surgical removal, while seven were managed with antireflux treatment and vocal therapy, without medication. In the first group, cure was seen in 50% after one or two surgeries; in the second group, the cure rate was 57%; there were no significant differences between them. For Karkos et al.^15^ conservative antireflux treatment is more effective than surgical removal in recurrent laryngeal granulomas. Fink et al.^21^ highlight the efficacy of botulinum toxin in the arytenoid muscle for granulomas refractory to surgical resection or treatment with proton pump inhibitors and/or vocal therapy. Sun et al.^22^ recommend the use of zinc sulfate in cases refractory to other treatments.

The great diversity of treatment modalities for post-intubation granulomas reflects the absence of consensus in the literature regarding the ideal treatment for the prevention of recurrence. The objective of this study was, therefore, to compare the effectiveness of the treatments found in the literature for laryngeal granulomas resulting from endotracheal intubation, in relation to resolution, rate of recurrence, and time to resolution.

## Materials and methods

Systematic review of studies that compared treatments of laryngeal granulomas secondary to endotracheal intubation.

### Criteria for considering studies for this review

Studies: randomized clinical trials (RCTs) or non-randomized controlled studies or observational studies (with at least 5 patients undergoing intervention) that analyze any type of treatment for laryngeal granuloma secondary to endotracheal intubation. Case reports and studies with less than five patients were excluded.

Participants: Patients with laryngeal granuloma secondary to endotracheal intubation.

Interventions: Conservative (proton pump inhibitors or oral H2 antagonists; antireflux measures; inhaled, oral or intralesional steroids; oral zinc sulfate; preventive voice care; speech therapy; oral antibiotics; topical botulinum toxin) or surgical procedures (conventional; fibroscopy or laser).

Outcomes: The primary outcome was resolution of the granuloma; secondary outcomes were recurrence after primary treatment, after secondary treatment, and duration of treatment for granuloma resolution.

### Search methods for studies identification

Electronic searches: Pubmed (1966 to January 2017); Embase (1980 to January 2017); Lilacs (www.bireme.br/) (1982 to January 2017); Cochrane database (1993 to January 2017); www.clinicaltrials.gov (assessed January 2017). A comprehensive search strategy was used: (Laryngeal Granulomas or Laryngeal Granuloma or Granuloma of Larynx or Larynx Granuloma or Larynx Granulomas or Vocal Folds Granuloma or Vocal Fold Granuloma or Vocal process granuloma) AND (Botulinum Toxins or Botulinum Toxin or Clostridium botulinum Toxins or Botulin or Surgery or operative therapy or operative procedures or invasive procedures or operations or Mitomycins or Lasers or Laser or Q-Switched Lasers or Q Switched Lasers or Q-Switched Laser or Pulsed Lasers or Pulsed Laser or Continuous Wave Lasers or Continuous Wave Laser or Masers or Maser or Zinc Sulfate or Zincteral or Heptahydrate Zinc Sulfate or Prilosec or Omeprazole Sodium or Omeprazole or Omeprazole magnesium or H 168-68 or H 168 68 or H 16 or Proton Pump Inhibitors or Inhibitors, Proton Pump). The search strategy was adapted for each database in order to achieve more sensitivity. There was no restriction concerning language or publication status. The references of relevant publications found by the search were screened for further studies and experts in the field were also contacted.

### Data collection and analysis

Studies selection: Two review authors (CR, RHGM) independently examined titles and abstracts in order to remove irrelevant reports; retrieved full-text copies of the potentially relevant reports; identified multiple reports from the same study by checking authors’ names, location and setting, details of the intervention, date and duration of the study; examined full-text reports for compliance with eligibility criteria; if necessary corresponded with authors in order to clarify any questions related to the study; and made a final decision on study inclusion.

Data extraction and management: Two review authors (CR, RHGM) independently extracted data from eligible studies and summarized them using a data extraction form. This summary contained the baseline characteristics of the study and included study type, age, gender, type of treatment, total number of participants, number of patients in each arm, follow-up, interventions and outcomes assessed.

Assessment of risk of bias in studies included: Was planned in RCTs, two review authors (CR, DCC) assessed each trial independently. In observational studies risk assessment for bias was not done due to lack of consensus for the application of this assessment in these studies, but they were considered as biased and subject to the effect of confounders. Disagreement was solved by consensus with participation of all authors.

Measures of treatment effect: Proportional meta-analysis was performed using the StatsDirect program, version 3.0.121. Forest plots were presented to summarize the data. Each horizontal line in the graph represented a study included in the meta-analysis. The combined total estimate was marked with a diamond at the bottom of the plot. Proportionality and 95% CI of the combined studies were presented. The presence of an overlap in the Confidence Intervals of the different interventions suggested a similar effect of the interventions on the outcome. No overlap suggested that there were distinct effects of the interventions studied. In order to quantify the inconsistencies of the studies used in the meta-analysis, the heterogeneity test *I*_2_ = [(*Q* − *df*)/*Q*] × 100% was used, where *Q* is the Chi-square and *df* the degree of freedom. We considered presence of substantial heterogeneity when *I*_2_ > 75%. Due to the heterogeneity between the studies, the meta-analysis random effect model was used.

For purposes of statistical analysis, the patients were divided into two subgroups according to the type of treatment used: Group 1 (Surgical ± associations); Group 2 (Surgical only).

The associations with surgical treatment were: antireflux therapy, speech therapy, antibiotic therapy, and steroids. As medical treatment only, antireflux therapy, speech therapy (or vocal preventive care), and oral or inhaled steroid therapy were considered.

Institutional review board approval was not necessary due to the nature of the study (Systematic review).

## Results

### Description of studies

#### Results of the search

**Figure 2:**
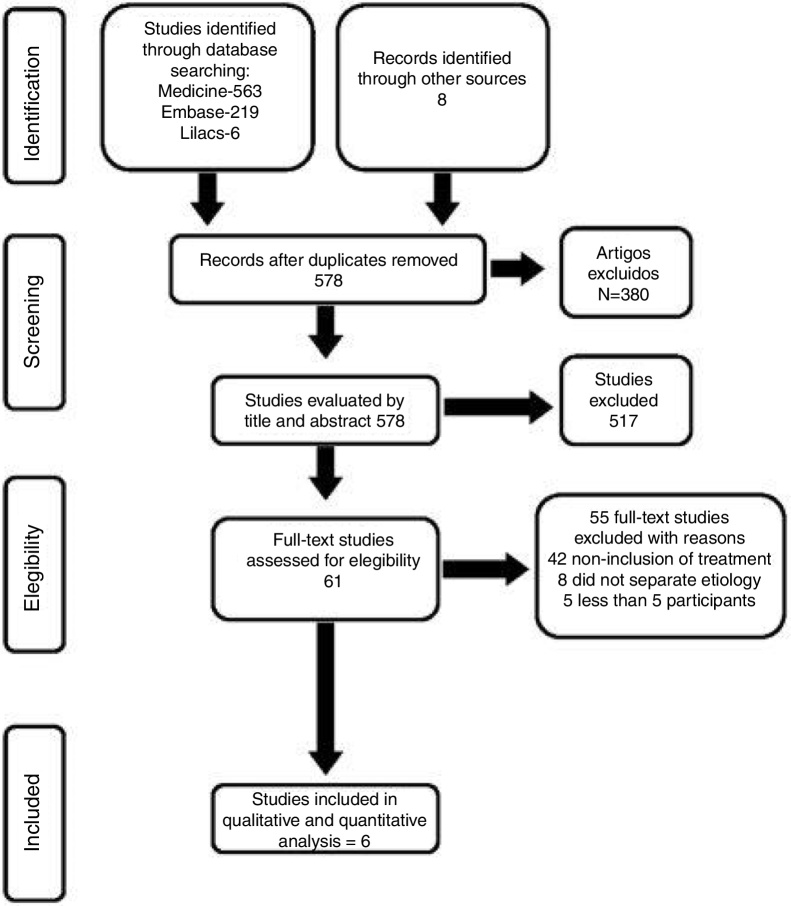
Flowchart showing the studies identified and evaluated during the review.

The search conducted in January 2017 recovered 563 studies in Medline, 219 in Embase, 6 in Lilacs, 0 in Cochrane and 8 in the manual search. After exclusion of duplicates and analysis of titles and abstracts, 61 articles were selected and obtained in full paper (Fig. 2). Of these, six studies considered the inclusion criteria previously defined, and 55 were excluded for the following reasons: 42 did not specifically include the treatment of intubation granulomas, eight did not distinguish results from those of the other types of granulomas, and five had less than five cases with intubation granulomas.

#### Studies included

The main characteristics of the selected studies were summarized in Table 1. The six included studies, with a total of 85 patients, were published in the period from 1999 to 2012, being prospective controlled non-randomized (*n* − 1), prospective uncontrolled (*n* − 1), and retrospective (*n* − 4). Two studies included only post-intubation granulomas,^24^, ^26^ and the others studied granulomas of different etiologies, but only post-intubation granulomas were included in the review.

**Table 1:** Studies characteristic.

Authors (year)	Study type site	Number of subjects	Gender (M/F)	Age (years)	Primary treatment	Outcome resolution after primary treatment	Outcome recurrence after primary treatment	Secondary treatment	Outcome recurrence after secondary treatment	Follow up (months)
de Lima Pontes et al. (1999)	Retrospective Cohort Brazil	12	9 F	Ø	Clinical *n* − 4	*n* − 12 (100%)	0	0	0	Ø
			6 M		Surgical n = 8					
Havas et al. (1999)	Retrospective Cohort Australia	14	Ø	Range age (30–86)	Clinical *n* − 6	Clinical *n* − 6 (100%)	Clinical 0	Antireflux therapy + Speech therapy *n* − 3	*n* − 1 (33%)	36
					Surgical *n* − 8	Surgical *n* − 5 (63%)	Surgical *n* − 3 (37%)			
Roh et al. (1999)	Non-randomized clinical trial South Korea	34	30 F 4 M	Range age (21–59)	Clinical *n* − 34	*n* − 26(76%)	*n* − 8 (24%)	Surgery ± laser	*n* − 2 (25%)	24
Hirano et al. (2002)	Uncontrolled clinical trial Japan	5	Ø	Ø	Surgical *n* − 5	*n* − 5 100%	0	0	0	16–72
Lin et al. (2008)	Case series China	12	7 F 5 M	Range age (32–73)	Surgical *n* − 12	*n* − 12 100%	0	0	0	14–43
Sun et al. (2012)	Case series China	8	Ø	Range age *n* − 2; 21–30 *n* − 2; 31–40 *n* − 1; 41–50 *n* − 1; 51–60 *n* − 2; 61–70	Surgical *n* − 8	*n* − 0 0%	*n* − 8 100%	Zinc sulfate	0	12

Ø, Unspecified data.

All articles were in the English language.

The number of patients in each study, gender, age group, type of intervention, outcomes studied, and follow-up time are depicted in Table 1.

### Types of intervention

Surgical treatment: The surgeries were conventional, KTP laser or fibroscopy with forceps that could be used several times.

Clinical treatment: antireflux therapy (either with a proton pump inhibitor – PPI or H2 inhibitor); speech therapy; anti-inflammatories; oral, inhaled or intralesional steroid; antibiotics; botulinum toxin and oral zinc sulfate.

### Types of outcomes

Six studies assessed resolution of the granuloma and recurrence after primary treatment, and three studies evaluated recurrence after secondary treatment. Five studies reported the time required for resolution of the granuloma.

### Studies excluded

The 55 studies excluded and the reasons for exclusion are depicted in Fig. 2.

#### Risk of bias in included studies

Although two of the studies were prospective, they were not randomized, and the other four were retrospective, therefore subject to the effect of confounders.

### Effects of interventions

#### Primary outcome

##### Granuloma resolution after primary treatment

**Figure 3:**
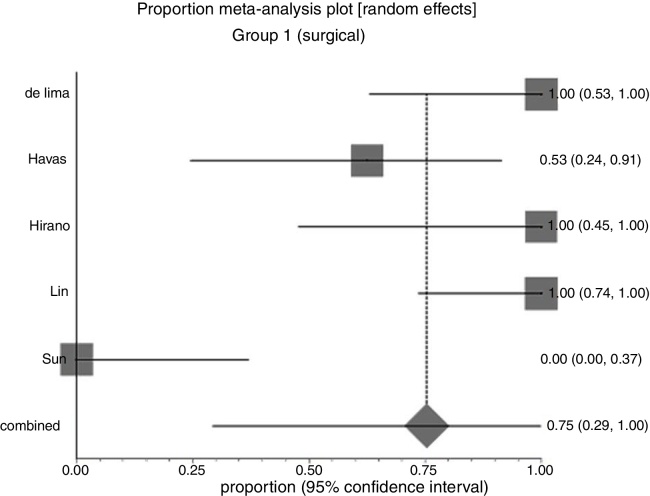
Forest plot for resolution after primary treatment with surgery ± associations.

**Figure 4:**
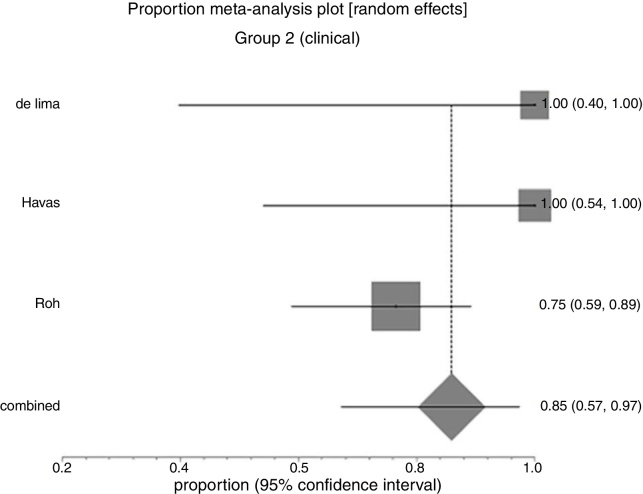
Forest plot for resolution after primary clinical treatment.

In Group 1 (surgery ± associations), including 41 patients, the chance of resolution of the granuloma was 75% (95% CI: 0.3–100%, *I*^2^ = 90%) (Fig. 3). In Group 2 (medical treatment), including 44 patients, the chance of resolution was 86% (95% CI: 67–97%) (Fig. 4).

Due to the high heterogeneity, the random effect was used for the meta-analysis.

**Figure 5:**
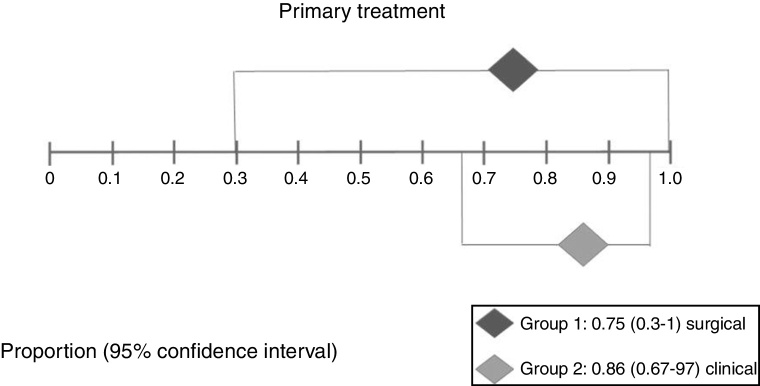
Interpretation of the meta-analysis for the outcome resolution after primary treatment. As there were overlapping confidence intervals, there was no statistically significant difference between the two groups.

Fig. 5 shows the results of the two interventions in relation to the outcome resolution after primary treatment, generated by statistical analysis (StatsDirect program). As there was overlap of the Confidence Intervals, there was no statistical difference between surgical and clinical treatments.

#### Secondary outcome

##### Relapse after primary treatment

**Figure 6:**
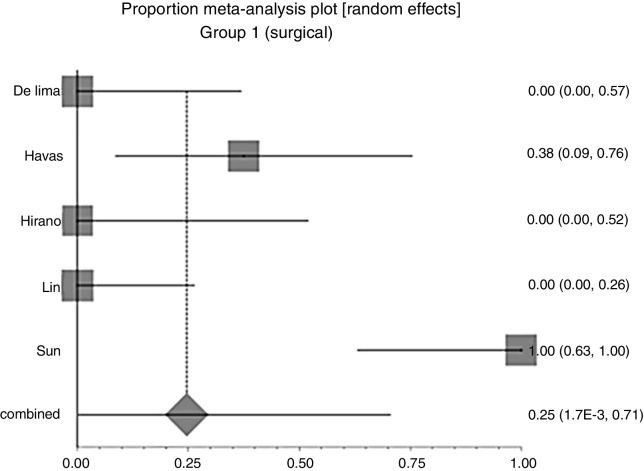
Forest plot for relapse after primary treatment with surgery ± associations.

**Figure 7:**
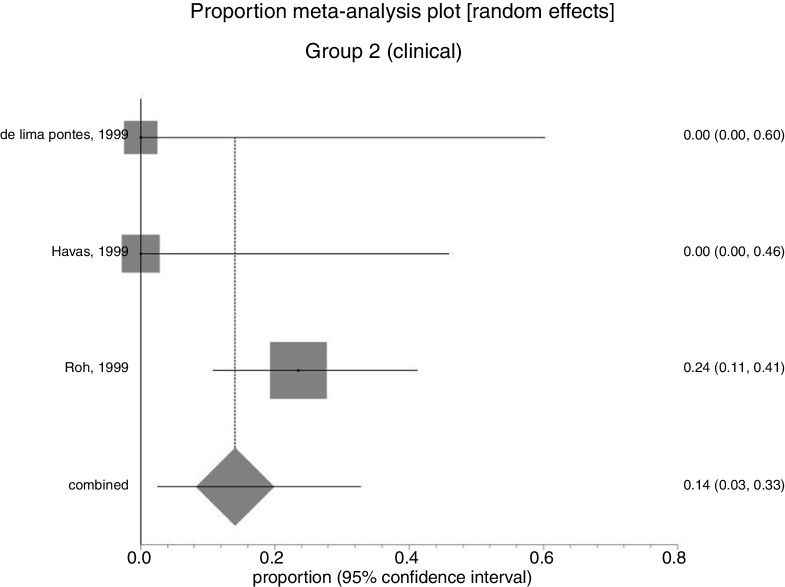
Forest plot for relapse after primary clinical treatment.

In Group 1 (surgery ± associations), including 41 patients (48%), the absolute recurrence risk was 25% (95% CI: 0.2–71%) (Fig. 6). In Group 2 (medical), including 44 patients (52%), the absolute risk of relapse was 14% (95% CI: 3–33%) (Fig. 7). Due to the high heterogeneity, for the meta-analysis, the random effect was used.

**Figure 8:**
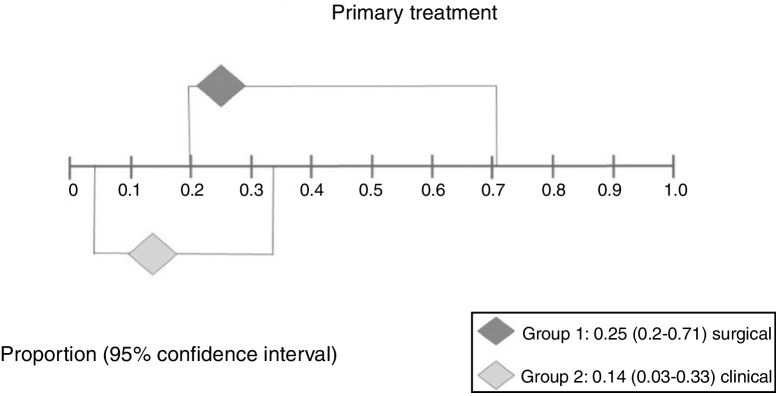
Interpretation of the meta-analysis for the outcome relapse after primary treatment. As there were overlapping confidence intervals, there was no statistically significant difference between the two groups.

Fig. 8 presents the results of the two interventions in relation to the outcome recurrence after primary treatment, generated by the statistical analysis. As there was overlap of the confidence intervals, there was no statistical difference between surgical and clinical treatments.

#### Recurrence after secondary treatment

Three studies, including 19 patients, analyzed the outcome recurrence after this new intervention. Of these, 3 (16%) presented new recurrence, achieving resolution in 16 patients.

Due to the low number of studies and/or patients, only the descriptive analysis of the data was done. Three patients who presented recurrence after surgical treatment were submitted to antireflux therapy associated with speech therapy, with resolution in two (recurrence rate 33%). Eight patients, also with recurrence after surgical treatment, used oral zinc sulfate, with complete resolution of all cases. Another eight patients, who presented recurrence after clinical treatment (vocal rest + ant-acids + oral anti-inflammatory ± oral steroid), were treated with conventional or laser surgery, with resolution of eight granulomas (25% relapse rate).

#### Treatment time required for the resolution of granulomas

The treatment time required to resolve the lesions varied, ranging from immediate following a procedure, as in the case of surgeries, to 23 months in the case of inhaled steroid (budesonide). Treatment with zinc sulfate took four to 12 weeks to resolve the lesions. The time of antireflux treatment, in turn, was not specified in all studies. The data presented were not subject to statistical analysis.

## Discussion

The recurrence rate of intubation granulomas renders the treatment of these lesions challenging. As we could verify in this review, few studies approach this topic with rigorous methodology and in many the information is incomplete and often the number of patients is small.

Analyzing the studies selected in this review, females (76%) and adults predominate, corroborating the findings of other authors.^1^, ^2^, ^3^ Even in post-intubation granulomas, gastroesophageal reflux is an important predisposing factor, and its treatment is the main clinical treatment indicated in the studies here listed. GERD is certainly a deleterious factor to the epithelization of the laryngeal mucosa damaged by intubation. Injuries to the laryngeal mucosa caused by exposure to gastric juice have been described for decades,^6^, ^9^ as well as the benefits of treatment with proton pump inhibitors in improving vocal symptoms and laryngeal lesions in patients with acid laryngitis. Proton pump inhibitors have been advocated in laryngeal granulomas, regardless of the etiology of the lesion.^14^, ^27^, ^28^

Vocal overuse is another factor predisposing to the formation of post intubation granulomas, as observed in two studies of this review, that included five patients.^1^, ^14^ Exaggerated phonation causes traumatic collision of the vocal folds, resulting in lesions of the arytenoid epithelium and delayed tissue healing.^1^

Choosing the ideal treatment for post-intubation granulomas has been a constant challenge for many specialists. Drug treatment alone is lengthy, therefore demotivating. According to Koufman,^28^ granuloma resolution with clinical treatment may take from six to eight months. Surgical treatment is faster, but involves anesthetic risks, local scars and recurrence, often being reserved for intractable cases. Even after surgery, clinical treatment is almost always initiated in order to reduce relapses, which can occur in 40–90%.^17^, ^20^, ^28^

In the papers selected in this review, the primary treatments included: antireflux therapy; speech therapy; anti-inflammatories; oral, inhaled or intralesional steroids; antibiotics; botulinum toxin and surgery. Secondary treatments included antireflux therapy; speech therapy; surgery and zinc sulfate. The meta-analysis of some of these studies did not indicate statistical difference between treatment modalities, probably due to the small number of patients, the high number of biases and the lack of detailed descriptions of the treatments, such as follow-up time and dose.

No revisions were found specifically about intubation granulomas. There is only one study about the treatment of laryngeal granulomas in general, without meta-analysis, and without the specification for granulomas due to intubation.^15^ According to these authors, reflux can be observed in up to 76% of patients with granulomas; therefore, antireflux therapy is the main treatment strategy for laryngeal granulomas, and surgery should be reserved only for therapeutic failures, airway obstruction or diagnostic doubt.^15^

The result of the surgical treatment does not seem to be influenced by the technique used (conventional, fibroscopy or laser) and was the modality of treatment with shorter resolution time. However, due to the high relapse rate, many authors emphasize the need for endoscopic follow-up of these patients for several months.^27^, ^28^, ^29^, ^30^

The studies included here demonstrated that the recurrence rates decrease when surgery is associated to other treatments. Botulinum toxin has been advocated along with surgery for granulomas of different causes.^19^, ^20^, ^21^, ^31^, ^32^ Among the advantages of this type of treatment we find no need for general anesthesia and minimum side effects.^19^

The results of this review indicated that there is no evidence as to the best treatment for laryngeal granulomas secondary to endotracheal intubation. The small number of patients in each study is responsible for large confidence intervals, which lead to indifference between interventions.

Regarding time to resolution of granulomas, surgical treatment is the fastest of all types of treatment, since it resolves immediately; however, recurrence is similar to the other treatments. Although inhaled budesonide is the treatment that has taken a longer time to resolve the lesions, it should be considered as an option for the treatment of laryngeal granulomas, since it has low morbidity. Zinc sulfate is a promising treatment, still little studied, which is also in the range of possibilities.

Regarding the applicability of the evidence, the meta-analysis performed in relation to the outcome resolution and recurrence of the granuloma after treatment, be it primary or recurrent, resulted in inconclusive data. There was no difference between the treatments analyzed due to the huge confidence intervals between the interventions, which inevitably lead to their overlap. As most of the included studies were considered to be at high risk of bias, the quality of the evidence is poor. Regarding the potential biases in the review process, we are confident that the extensive literature search used in this review, added to the manual search, has captured most of the relevant literature published, minimizing the likelihood of loss of relevant studies.

## Conclusions

There is no high quality evidence to prove the effectiveness of any treatment for laryngeal granulomas resulting from endotracheal intubation.

### Implications for research

Systematic reviews of surgical interventions are difficult to perform, since randomized studies for the surgical area are rare. Comparison of clinical and surgical interventions is even more difficult through randomized clinical trials. Therefore, we believe that controlled, even non-randomized, multicenter studies are needed with each center performing the intervention they have the most expertise, with a neutral outcome assessor. In addition, it is necessary to standardize the data described.

### Implications for practice

Since evidences that favor one or other treatment are inexistent, any treatment may be used according to the experience of the group involved.

## Conflicts of interest

The authors declare no conflicts of interest.
